# What limits the utilization of health services among china labor force? analysis of inequalities in demographic, socio-economic and health status

**DOI:** 10.1186/s12939-017-0523-0

**Published:** 2017-02-02

**Authors:** Liming Lu, Jingchun Zeng, Zhi Zeng

**Affiliations:** 1 The Second Affiliated Hospital of Guangzhou University of Chinese Medicine, Guangdong Provincial Hospital of Chinese Medicine, 111 Dade Road, Guangzhou, 510120 China; 2 Guangzhou University of Chinese Medicine, Guangzhou, 510405 China; 3 Hunan Provincial Maternal and Child Health Care Hospital, #53 Xiangchun Road, Changsha, 410008 China

**Keywords:** China, Utilization of health service, Labor force, Inequalities

## Abstract

**Background:**

Inequalities in demographic, socio-economic and health status for China labor force place them at greater health risks, and marginalized them in the utilization of healthcare services. This paper identifies the inequalities which limit the utilization of health services among China labor force, and provides a reference point for health policy.

**Methods:**

Data were collected from 23,505 participants aged 15 to 65, from the 2014 China Labor Force Dynamic Survey (a nationwide cross-sectional survey covering 29 provinces with a multi-stage cluster, and stratified, probability sampling strategy) conducted by Sun Yat-sen University. Logistic regression models were used to study the effects of demographic (age, gender, marital status, type of *hukou* and migration status), socio-economic (education, social class and insurance) and health status (self-perceived general health and several chronic illnesses) variables on the utilization of health services (two-week visiting and hospitalization during the past 12 months). Goodness of fit was assessed using Hosmer-Lemeshow test. Discrimination ability was assessed based on the area under the receiver operating curve (AUC).

**Results:**

Migrants with more than 1 (OR 2.80, 95% CI 1.01 ~ 7.82) or none chronic illnesses (OR 1.26, 95% CI 1.01 ~ 7.82) are more likely to be two week visiting to the clinic than non-migrants; migrants with none chronic illnesses (OR 0.61, 95% CI 0.45 ~ 0.82) are less likely to be in hospitalization during the past 12 months than non-migrants. Female, elder, hukou of non-agriculture, higher education level, higher social class, purchasing more insurance and poorer self-perceived health were predictors for more utilization of health service. More insurance benefited more two-week visiting (OR 1.12, 95% CI 1.06 ~ 1.17) and hospitalization during the past 12 months (OR 1.12, 95% CI 1.07 ~ 1.18) for individuals with none chronic illness but not ≥1 chronic illnesses. All models achieved good calibration (Hosmer-Lemeshow test’s P range of 0.258-0.987) and discrimination (AUC range of 0.626-0.725).

**Conclusions:**

This study has shown that there are inequalities of demographic, socio-economic and health status in the utilization of health services for China labor force. Prudent health policy with equitable utilization of health services eliminating mentioned inequalities should be a priority in shaping China’s healthcare system reform.

## Background

China has experienced dramatic industrialization, urbanization, and economic growth over the last three decades [1]. As the important drivers of economic growth, a huge number of labor force are still required in China. The term of labour force means the labour pool in employment and is generally used to describe those working for a single company or industry. Most of the labor force are migrants. By 2014, China’s migrant population had reached 253 million [2]. Unfortunately, the rapid growth of available wealth and labor welfare has not been distributed evenly across the labor force, leading to the wealth gap and increased disparity in health service utilization among different types of labor force [2]. Health facilities and services distribute unevenly among different provinces and the eastern is generally better than the central and western [3, 4]. It is reported that the Gini coefficient was higher than 0.5 in terms of local governments devoting outlay for the community health service [5], which means the vulnerable population of the labor force face more barriers to access the health service and are more vulnerable to health problems compared to the general population, due to their low levels of employment benefits and social security [6].

How to improve the equitable utilization of health services is still a significant concern for labor force and an imminent policy challenge for sustainable regional economic development. Equity in health service utilization are important objectives for the healthcare system that are increasingly attracting attention in China [7]. Reducing inequality has been widely considered a major aim of health care policies in China. Overall, there is substantial health inequality within the Chinese labor population, especially in the vulnerable populations [8]. However, the utilization of health service among labor force is rarely studied in China. There lacks a knowledge base about how to promote the utilization of health service among labor force.

Previous studies have demonstrated that the primary contributors towards the utilization of health service: 1) demographic determinants, including gender, age, marital status, migrant status, and type of *hukou* [9–12], 2) socio-economic determinants, including education level, social class and insurance [13–18], and 3) health status such as self-perceived health and chronic illnesses [19, 20]. Use of outpatient care over a two week period and use of inpatient care over a 12 month period are two comon measurements of health service utilization [21] included in our study.

This study uses a nationally representative sample to explore the status of health service utilization, examine its associated factors and determine the relative importance of these factors among labor force. Results of the study would help understand the demographic, socio-economic and health-related inequalities to improve the utilization of health service among labor force.

## Methods

### Data and sampling

The data were derived from the 2014 Chinese Labor Dynamic Survey (CLDS), which was the second wave of a nationally representative panel survey [22]. CLDS was (a nationwide cross-sectional survey covering 29 provinces excluding Tibet and Hainan with a multi-stage cluster, and stratified, probability sampling strategy) conducted by Sun Yat-sen University. The second wave was conducted in the same communities as the first wave (applied in year 2012). Of a total of 23,594 respondents in the second wave, 10,053 were follow up samples from the first wave, and 13,541 were newly added participants. We only used the data of the second wave and deleted respondents with more than 15% data missing for one variable in our analysis (the proportion of missingness was 0.38%), finally 23,505 respondents were included in our analysis.

### Availability of data and materials

The data used in our study can be applied and obtained from the 2014 CLDS of Center for Social Survey, Sun Yat-sen University (available online: http://css.sysu.edu.cn/Data). More sampling process of this survey can be found in one previous published report (https://www.ncbi.nlm.nih.gov/pubmed/26215980).

### Variables and measurement

#### The utilization of health service

The following two dependent variables were included and represented by the two-week visiting to the clinic (a person visited the clinic at least one time within two weeks) and by admissions to hospital during the past 12 months when the respondent was sick or injury. They were dichotomized to 0–1 values (0-non-use and 1-use).

#### Demographic factors

Demographic characteristics used in this paper included age group (categorized into 15 year age groups: “15–29”, “30–44”, “45–59” and “≥60”), gender (“female” and “male”), marital status (“single”, “married: first marriage”, “married: non-first marriage”, “divorced” and “widowed”), migrant (“Yes” and “not”), and type of *hukou* (“agriculture” and “non-agriculture”).

#### Socio-economic factors

The variables reflecting socio-economic position were education level of the respondent (“primary school or below”, “junior secondary school”, “senior secondary school” and “junior college and above”), social class (and a 5-point Likert response scale of “poorest class”/“poorer class”/“middle class”/“richer class”/“richest class” was used for items of social class) and the number of purchasing insurance (a continuous variable).

#### Health status

Health status included self-perceived health (categorized into 3 groups: “poor”, “average” and “good”), and the number of chronic illnesses (a continuous variable) which comprised tuberculosis, asthma, chronic obstructive pulmonary disease, hypertension, coronary heart disease, stroke, hepatitis, diabetes, genetic diseases and cancer.

### Statistical analysis

Firstly, the sampling process considered the investigation design effect, therefore we set out sampling parameters and adjusted the systematic error caused by sampling design. The weighted means, ratio and standard errors of variables were reported in descriptive results.

For another, to enhance the information provided by the dependent variables (Y1 = two-week visiting or Y2 = hospitalization during the past 12 months), we stratified the sample by the number of chronic conditions (no chronic illness, one chronic illness, more than 1 chronic illnesses). A logistic regression was applied to assess the influences of migration with different number of chronic conditions on the utilization of health services. Goodness of fit between the observed and predicted outcomes of the logistic model were assessed based on the Hosmer-Lemeshow test and its discrimination ability was assessed based on the area under the receiver operating curve (AUC). The probability, *P* ≤ 0.05 was considered to indicate statistical significance. We used “mean replacement method” to handle the missing data (less than 15% data missing for one variable). “Mean replacement method” involves replacing any missing value with the mean of that variable for all other cases, which has the benefit of not changing the sample mean for that variable.

All of the descriptive statistical analysis and statistical inference were performed using Stata statistical software version 11.0 (StataCorp LP, College Station, TX, U.S.).

## Results

### Descriptive analysis

The unweighted and weighted estimates of demographic, socio-economic and health related information of study participants were reported in Table 1. Nearly 48.0% of the participants were male and less than 45 years old; nearly 70% were educated below senior secondary school education and had the *hukou* of agriculture; 82.0% were married; more than 90% believed they were at or below middle social class; in average, they bought 1–2 insurance and had no more than one chronic illnesses; and 62.0% perceived themselves in good health.

**Table 1:** Distribution Difference of Demographic, Socio-economic and Health Status in the Utilization of Health Services among China Labor Force

Variables	Unweighted estimates		Weighted estimates^a^	
	N (%) or mean	Standard error	Proportions (%) or mean	Standard error
Demographic Factors				
Gender				
Male	11281 (48.0)	0.0033	50.9	0.0048
Female	12224 (52.0)	0.0033	49.1	0.0048
Age (years)	43.95	0.0944		
Marital Status				
Single or divorced	4240(18.0)	0.0025	24.4	0.0081
Married	19265(82.0)	0.0025	75.6	0.0081
Migrant				
Yes	2141 (9.1)	0.0019	10.5	0.0129
No	21364 (90.9)	0.0019	89.5	0.0129
type of *hukou*				
Agriculture	16467 (70.1)	0.0030	72.2	0.0298
Non-agriculture	7038 (29.9)	0.0030	27.8	0.0298
Socio-economic Factors				
Education Level				
Primary school or below	8535 (36.3)	0.0031	23.6	0.0457
Junior secondary school	7707 (32.8)	0.0031	46.8	0.0457
Senior secondary school	4161 (17.7)	0.0025	18.0	0.0457
Junior college and above	3102 (13.2)	0.0022	11.6	0.0457
Social class				
Poorest class	2866 (12.2)	0.0021	12.5	0.0210
Poorer class	6777 (28.8)	0.0030	29.4	0.0210
Middle class	11694 (49.8)	0.0033	49.7	0.0210
Richer class	1907 (8.1)	0.0018	7.4	0.0210
Richest class	261 (1.1)	0.0007	1.0	0.0210
Number of insurance	1.76	0.0099	1.63	0.0494
Health status				
Self-perceived health				
Poor	2992 (12.7)	0.0022	8.9	0.0166
Average	5947 (25.3)	0.0028	22.8	0.0166
Good	14566 (62.0)	0.0022	68.4	0.0166
Number of chronic illnesses	0.15	0.0029	0.10	0.0054

^a^Survey design effects (strata, cluster, family, and individual weight) were adjusted in the mean and proportion estimations

Among 23,505 respondents, 1119 (4.8%) and 1367 (5.8%) underwent the experience of two-week visiting to the clinic and hospitalization during the past 12 months, respectively.

### The association between chronic illnesses and the utilization of health service

From Table 2, migrants with more than 1 (OR 2.80, 95% CI 1.01 ~ 7.82) or none chronic illnesses (OR 1.26, 95% CI 1.01 ~ 7.82) were more likely to be two week visiting to the clinic than non-migrants; migrants with none chronic illnesses (OR 0.61, 95% CI 0.45 ~ 0.82) are less likely to be in hospitalization during the past 12 months than non-migrants. Female, elder, hukou of non-agriculture, higher education level, higher social class, purchasing more insurance and poorer self-perceived health were predictors for more utilization of health service. More insurance benefited more two-week visiting (OR 1.12, 95% CI 1.06 ~ 1.17) and hospitalization during the past 12 months (OR 1.12, 95% CI 1.07 ~ 1.18) for individuals with none chronic illness but not ≥1 chronic illnesses. All models achieved both calibration (Hosmer-Lemeshow test’s *P* range of 0.258-0.987) and discrimination (AUC range of 0.626-0.725).

**Table 2:** ORs (95% CIs) of Health Services Utilization according to Chronic Conditions

	Two-week visiting Number of chronic illnesses			Hospitalization during the past 12 months Number of chronic illnesses		
	0	1	2+	0	1	2+
Demographic Factors						
Gender						
Male	-	-	-	-	-	-
Female	1.10 (0.95 ~ 1.27)	**1.42** (1.05 ~ 1.91)*	1.17 (0.70 ~ 1.95)	**1.22** (1.05 ~ 1.42)**	1.02 (0.82 ~ 1.28)	1.18 (0.79 ~ 1.77)
Age (years)	**0.87** (0.79 ~ 0.95)**	0.98 (0.81 ~ 1.20)	**0.62** (0.41 ~ 0.94)*	0.94 (0.86 ~ 1.03)	1.05 (0.90 ~ 1.22)	0.97 (0.70 ~ 1.35)
Marital Status						
Single or divorced	-	-	-	-	-	-
Married	0.86 (0.70 ~ 1.05)	1.62 (0.93 ~ 2.81)	0.89 (0.39 ~ 2.01)	1.16 (0.93 ~ 1.44)	1.06 (0.73 ~ 1.53)	1.04 (0.54 ~ 2.02)
Migrant						
No	-	-	-	-	-	-
Yes	**1.26** (1.01 ~ 1.60)*	0.91 (0.48 ~ 1.75)	**2.80** (1.01 ~ 7.82)*	**0.61** (0.45 ~ 0.82)**	0.86 (0.52 ~ 1.43)	0.70 (0.25 ~ 2.00)
Type of *hukou*						
Agriculture	-	-	-	-	-	-
Non-agriculture	0.91 (0.75 ~ 1.11)	1.00 (0.69 ~ 1.46)	0.66 (0.36 ~ 1.22)	0.97 (0.80 ~ 1.19)	1.31 (0.99 ~ 1.72)	**2.25** (1.42 ~ 3.55)**
Socio-economic Factors						
Education level	**0.86** (0.78 ~ 0.95)**	1.02 (0.83 ~ 1.25)	0.93 (0.65 ~ 1.32)	0.91 (0.82 ~ 1.00)	0.94 (0.80 ~ 1.10)	0.86 (0.65 ~ 1.13)
Social class	0.96 (0.88 ~ 1.05)	1.05 (0.89 ~ 1.24)	0.91 (0.70 ~ 1.20)	1.06 (0.97 ~ 1.16)	**1.17** (1.03 ~ 1.33)*	0.90 (0.72 ~ 1.12)
Number of insurance	**1.12** (1.06 ~ 1.17)**	1.08 (0.96 ~ 1.22)	1.22 (0.99 ~ 1.49)	**1.12** (1.07 ~ 1.18)**	0.95 (0.86 ~ 1.05)	0.97 (0.82 ~ 1.15)
Health status						
Self-perceived health	**0.34** (0.31 ~ 0.38)**	**0.40** (0.31 ~ 0.50)**	0.66 (0.42 ~ 1.03)	**0.36** (0.33 ~ 0.40)**	**0.49** (0.42 ~ 0.57)**	**0.58** (0.41 ~ 0.83)**
Hosmer-Lemeshow test’s *P*	0.987	0.485	0.326	0.338	0.503	0.258
AUC (95% CI)	0.725 (0.719-0.731)	0.688 (0.670-0.706)	0.652 (0.608-0.693)	0.720 (0.714-0.726)	0.654 (0.634-0.672)	0.626 (0.582-0.668)

*CI* confidence interval, *AUC*, area under the receiver operating curve, *P ≤ 0.05, **P ≤ 0.01

The data in bold meant the result was significant in statistics

## Discussion

Our results showed that significant difference in gender, migrant status, social class, insurance purchasing, self-perceived health and chronic illnesses suffering were the key predictors influencing the utilization of health service among China labor force by a nationally representative survey. The significant predictors for two-week visiting to the clinic and hospitalization during the past 12 months were similar.

Our findings regarding the impact of demographic factors on the health services utilization were consistent with previous studies that female were more likely to use health service [3, 23]. What can be explained was that female was sensitive to and had the higher awareness of health problems and symptoms than male [3, 23]. Men were more inclined to self-sustainable and self-medication when they felt unwell.

For the socio-economic factors, respondents of advantaged classes and purchasing more insurance were more likely to access the health service, which were consistent with the results of previous studies [24, 25]. Compared to respondents with agriculture hukou, non-agriculture with +2 chronic conditions benefited more hospitalization. People with non-agriculture hukou have a higher income and social status than agriculture. These findings could be explained by the fact that people with advantaged classes, more insurance and non-agriculture hukou were more capable to afford the medical expenditure and had a privileged position concerning health service. However, previous studies on health service inequalities also showed inconsistent findings. Some studies found that the frequency of health service utilization increased with disadvantaged social class [26, 27]. Other studies found no association between social class inequalities and the utilization of health service [28, 29]. The different results of the relationship between socio-economic factors and the utilization of health service could be explained by different social insurance systems and accessibility mechanism of health service in different countries [3].

We believed our results might provide some information to explain the appropriateness and barriers of health service utilization in China, which required to be confirmed with more robust study designs (longitudinal, time-series analysis, etc.). It was noteworthy that migrants with more chronic illnesses may require care of two week visiting more often than non-migrants and migrants faced more barriers to access the hospitalization, as they were often deprived of local public health services and medical insurance schemes due to the household registration system in China [30]. For the low levels of employment benefits, social security and high mobility, migrants are particularly vulnerable to health problems [31]. Many migrants with disadvantaged social classes and limited insurance in China were more inclined to seek cheap and simple health service in clinic around their communities rather than in hospital, as they were unwilling to pay the expensive medical examination and therapies in hospital. For the health status factors, echoing with previous published studies [32–34], self-perceived health and number of chronic illnesses were two measures of health service need and also important contributors to the utilization of health service. That was, poorer self-perceived health status and more chronic illnesses prompts people to seek health service. However, our results did not observe any obvious barriers migrants with one or more chronic illnesses faced to access the hospitalization. The reason was that many migrants with more chronic illnesses may return home due to their weak health and inability to afford the work in cities, which led to the small number of migrants with more chronic illnesses included in the investigation and insufficient statistical power to get the significant results. For another, we thought the variable of age and sex might be the main confounding factors affected the relationship between chronic illnesses and the utilization of health service. Thus, we performed a complemented sensitive analysis by stratifying the sample by “the number of chronic conditions, age (>50 and ≤50) and migration status”, OR “the number of chronic conditions, sex (female and male) and migration status”, respectively. We observed all logistic regressions got the smilar results and we were confident to say that our results were stable without overestimation and underestimation of some variables. Thus, we built the model by including all the covariates to control the possible baseline and socio-economic factors and got the more precise estimation with good calibration (Hosmer-Lemeshow test’s *P* range of 0.258-0.987) and discrimination (AUC range of 0.626-0.725).

A number of policy implications can be drawn from our study to improve the utilization of health service for China labor force. Firstly, priority should be given to satisfy the needs of the vulnerable (eg. migrants, disadvantaged classes) in China’s health care reform. Secondly, governments and employers should ensure the enabling resources to be provided to the labor force. It is imperative to provide labor force with effective security benefits such as labor contracts and insurances, especially for individuals with chronic illnesses. Thirdly, the clinic in China should provide better service of chronic disease management and treatment for migrants. The government and society should make some efforts to reduce the barriers of access to the hospitalization for migrants. Understanding of existing inequalities in utilization of health services benefits us making more useful and practical policy targeted the vulnerable mentioned above. However, some limitations should be born in mind. First, dichotomized dependent variables can't be used as proxies of quality of care or guideline-concordant treatment by themselves. But, the identification of coherent groups (stratified the sample by the number of chronic conditions in our study) to compare can help identify more important associations that can be intervened on. Second, variables’ collecting is based on self-reported method and may be measured with errors; our study is cross-sectional, such that causality cannot be determined. Third, health service utilization was also determined by the accessibility of health services in terms of geography, cultural, administrative barrier besides the socio-economic factors and quality issues of our study. It is necessary to consider more associated factors in future.

## Conclusions

In conclusion, this study has shown that there are inequalities of demographic, socio-economic and health status in the utilization of health services for labor force in China. Prudent health policy with equitable utilization of health services eliminating mentioned inequalities should be a priority in shaping China’s healthcare system reform.

## Abbreviations

CI: Confidence interval.
CLDS: Chinese labor dynamic survey
OR: Odds ratio
